# Humoral and Cellular Responses to COVID-19 Vaccines in SARS-CoV-2 Infection-Naïve and -Recovered Korean Individuals

**DOI:** 10.3390/vaccines10020332

**Published:** 2022-02-18

**Authors:** Ji-Young Hwang, Yunhwa Kim, Kyung-Min Lee, Eun-Jeong Jang, Chang-Hoon Woo, Chang-Ui Hong, Seok-Tae Choi, Sivilay Xayaheuang, Jong-Geol Jang, June-Hong Ahn, Hosun Park

**Affiliations:** 1 Department of Microbiology, College of Medicine, Yeungnam University, Daegu 42415, Korea; generous81@yu.ac.kr (J.-Y.H.); linbi@ynu.ac.kr (Y.K.); blackxg@yu.ac.kr (K.-M.L.); lemon3321@yu.ac.kr (E.-J.J.); tjrxopk@yu.ac.kr (S.-T.C.); sivilayxayaheuang@yu.ac.kr (S.X.); 2 Department of Pharmacology, College of Medicine, Yeungnam University, Daegu 42415, Korea; changhoon_woo@yu.ac.kr (C.-H.W.); ahnton9601@ynu.ac.kr (C.-U.H.); 3 Division of Pulmonology and Allergy, Department of Internal Medicine, College of Medicine, Yeungnam University, Daegu 42415, Korea; jang83@ynu.ac.kr (J.-G.J.); fireajh@yu.ac.kr (J.-H.A.); 4 Immunogenicity Evaluation Laboratory, Clinical Trial Center, Yeungnam University Medical Center, Daegu 42415, Korea

**Keywords:** COVID-19, vaccine, antibody, ELISA, pVNT_50_, ELISpot

## Abstract

In the face of a global COVID-19 vaccine shortage, an efficient vaccination strategy is required. Therefore, the immunogenicity of single or double COVID-19 vaccination doses (ChAdOX1, BNT162b2, or mRNA-1273) of SARS-CoV-2-recovered individuals was compared to that of unvaccinated individuals with SARS-CoV-2 infection at least one year post-convalescence. Moreover, the immunogenicity of SARS-CoV-2-naïve individuals vaccinated with a complete schedule of Ad26.CoV2.S, ChAdOX1, BNT162b2, mRNA-1273, or ChAdOX1/BNT162b2 vaccines was evaluated. Anti-SARS-CoV-2 S1 IgG antibody (S1-IgG), pseudotyped virus-neutralizing antibody titer (pVNT_50_), and IFN-γ ELISpot counts were measured. Humoral immune responses were significantly higher in vaccinated than in unvaccinated recovered individuals, with a 43-fold increase in the mean pVNT_50_ values. However, there was no significant difference in the pVNT_50_ and IFN-γ ELISpot values between the single- and double-dose regimens. In SARS-CoV-2-naïve individuals, antibody responses varied according to the vaccine type: BNT162b2 and mRNA-1273 induced similar levels of S1-IgG to those observed in vaccinated, convalescent individuals; in contrast, pVNT_50_ was much lower in SARS-CoV-2-naïve vaccinees than in vaccinated recovered individuals. Therefore, a single dose of ChAdOX1, BNT162b2, or mRNA-1273 vaccines would be a good alternative for recovered individuals instead of a double-dose regimen.

## 1. Introduction

Since the first case of severe acute respiratory syndrome coronavirus 2 (SARS-CoV-2) infection was reported in December 2019, there have been more than 318 million confirmed cases of coronavirus disease (COVID-19) as of January 2022, including 5.5 million deaths worldwide [1]. The COVID-19 pandemic accelerated the development and production of several vaccine platforms, such as mRNA, viral vector, inactivated, subunit, and DNA vaccines. Until now, the WHO has approved ten COVID-19 vaccines for emergency use, i.e., two mRNA vaccines—BNT162b2 (Pfizer/BioNTech) and mRNA-1273 (Moderna)—three non-replicating viral vector vaccines—ChAdOX1 (Oxford/AstraZeneca), Covishield (Oxford/AstraZeneca/Serum Institute of India), and Ad26.COV2.S (Janssen)—three inactivated vaccines—Covaxin (Bharat Biotech), CoronaVac (Sinovac), and BBIBP-CorV (Sinopharm)—and two protein subunit vaccines—NVX-CoV2373 (Novavax) and COVOVAX (Novavax/Serum Institute of India) [2]. Most countries implemented mass vaccination campaigns to achieve herd immunity and reduce morbidity and mortality. With the fourth COVID-19 wave progressing worldwide and the increasing threat of new variants, extensive vaccination programs should be implemented to maximize efficient vaccine provision with limited vaccine resources. Most recovered patients already have some immunity against SARS-CoV-2, although it is insufficient to protect against variants. The currently available COVID-19 vaccines designed against the spike protein of wild-type SARS-CoV-2 effectively protect against severe COVID-19 and death caused by the variants [3,4,5,6]. However, the imbalance between the demand and supply of COVID-19 vaccines is a serious concern, especially in low-income countries. Additional doses have been administered to individuals who have received a complete vaccine dose in high-income countries. Only 13.6% of the population had received at least a single vaccine dose in Africa as of 5 January 2022 [7]. Therefore, strategies for effective vaccine provision should be considered.

Recently, several reports have demonstrated that recovered individuals exhibit better immune responses, including cross-protective immunity against variants, than infection-naïve individuals with a single dose of an mRNA COVID-19 vaccine [8,9,10,11,12,13,14,15]. However, only limited data are available on viral vector vaccines, such as ChAdOX1, in SARS-CoV-2-recovered individuals [16,17,18].

In order to evaluate the humoral and cellular immune responses elicited by ChAdOX1, BNT162b2, and mRNA-1273 vaccines in recovered individuals, we performed age-, sex-, and initial symptom onset (ISO)-matched case–control studies between a single-dose-vaccinated group and an unvaccinated group of recovered individuals. Moreover, we compared the immunogenicity of mRNA and viral vector vaccines in SARS-CoV-2-naïve individuals.

## 2. Materials and Methods

### 2.1. Participants and Study Design

To evaluate vaccine-induced immunity in recovered and infection-naïve healthy individuals, we selected 52 participants from the YUMC-COVID-R02 study (IRB No. YUMC-2020-04-009), an ongoing longitudinal immunogenicity evaluation study of COVID-19-convalescent individuals, and 62 healthy SARS-CoV-2-naïve individuals were newly enrolled (IRB No. YUMC-2021-03-012). COVID-19-convalescent patients had been diagnosed using RT-PCR during the first wave of COVID-19 (from February 2020 to April 2020) [19]. They had been discharged from either hospitals or residential treatment centers and visited Yeungnam University Medical Center four times for blood sampling after recovery. Among them, 18 were vaccinated with one dose, and 17 were vaccinated with two doses of COVID-19 vaccines (ChAdOX1, BNT162b2, mRNA-1273, or ChAdOX1/BNT162b2 vaccines) between visits 3 and 4, at least one year after recovery, except for one individual. Convalescent individuals with a single dose or without vaccination were matched for age, sex, and number of days after ISO at time of blood collection and nested case-control analyses were performed. Blood samples were collected from SARS-CoV-2-naïve individuals within 1 week before vaccination and 4–5 weeks after the completion of the vaccination schedule. Plasma was stored at −80 °C, and peripheral blood mononuclear cells (PBMCs) were cryopreserved in liquid nitrogen until use.

### 2.2. Anti-SARS-CoV-2 IgG Enzyme-Linked Immunosorbent Assay (ELISA)

To detect anti-SARS-CoV-2 S1 and nucleocapsid protein (NCP) IgG antibodies, plasma samples were tested with EUROIMMUN ELISA SARS-CoV-2 IgG (EUROIMMUN AG, Lübeck, Germany) coated with recombinant SARS-CoV-2 spike protein (S1 domain) or NCP. ELISA was performed according to the manufacturer’s instructions. Optical density (OD) was measured at a wavelength of 450 nm with a reference wavelength of 620 nm using a microplate reader (Multiskan FC, Thermo Scientific, Waltham, MA, USA). The results were evaluated semiquantitatively by calculating the ratio between the extinction of the control or sample and that of the calibrator. The ratio was calculated using the following formula:

Ratio = Extinction of the control or sample/extinction of calibrate

Samples with a ratio of <0.8 were interpreted as negative, 0.8–<1.1 were borderline, and ≥1.1 were positive.

### 2.3. Pseudotyped Virus Neutralization Test (pVNT)

HEK-293T cells (ATCC, Manassas, VA, USA) were transfected with psPAX (Addgene #12260, Cambridge, MA, USA), pLV-Luc, and pcDNA3.1-SARS-CoV-2-Spike (Addgene #145032, Gene ID 43740568) using Solfect^TM^ transfection reagent (BIOSOLYX, Daegu, Korea). The plasmid pLV-Luc was constructed by PCR cloning with specific restriction enzyme primers (forward BamHI primer: 5′-ggcg ggcg GGA TCC accggtcgccacc ATG GAA GAT GAT GCC AAA AAC ATT AAG AAG-3′ and reverse SalI-primer: 5′-ggcg ggcg GTC GAC gcggccgct TTA CAC GGC GAT CTT GCC GCC C-3′). pLV-Luciferase was constructed by subcloning the firefly luciferase gene insert from pGL3 (Genbank U47295.2, Promega, Madison, WI, USA)—digested with BamHI and SalI (Takara, Shiga, Japan)—into the respective site in the pLV-eGFP vector (Addgene #36083). At 48 h post-transfection, the culture media (SARS-CoV-2 pseudovirus) were harvested. After centrifugation, supernatants were collected as pseudoviral particles. The SARS-CoV-2 pseudovirus was stored at −80 °C until use. The plasma samples were heat-inactivated for 30 min at 56 °C and serially diluted 2-fold in Dulbecco’s modified eagle medium (Lonza, Walkersville, MD, USA) supplemented with 10% heat-inactivated FBS (Gibco, Grand Island, NY, USA). Diluted plasma samples were mixed with an equal volume of SARS-CoV-2 pseudovirus for 1 h at 37 °C. One hundred microliters of diluted plasma/virus mixture, media/virus mixture (positive control), or medium alone (negative control) were inoculated into angiotensin-converting enzyme 2-(ACE2)-expressing HEK 293 T cells (GeneCopoeia, Rockville, MD, USA) in a 96-well clear-bottom white plate (Corning, Corning, NY, USA). After 48 h of infection, the supernatant was discarded. To each well, 20 µL of 1× passive lysis buffer (Promega) were added, followed by the addition of 100 µL of luciferase assay reagent (Promega). Luciferase activities of SARS-CoV-2 pseudovirus were detected using a Molecular Devices L-Max II Luminescence Microplate Reader (Molecular Devices, San Jose, CA, USA) and expressed as relative luminescence units (RLU). The inhibition rate (%) of samples was calculated as follows:

$$1-\frac{\left(\mathrm{mean}\;\mathrm{RLU}\;\mathrm{of}\;\mathrm{the}\;\mathrm{sample}-\mathrm{mean}\;\mathrm{RLU}\;\mathrm{of}\;\mathrm{the}\;\mathrm{negative}\;\mathrm{control}\right)}{\left(\mathrm{mean}\;\mathrm{RLU}\;\mathrm{of}\;\mathrm{the}\;\mathrm{positive}\;\mathrm{control}-\mathrm{mean}\;\mathrm{RLU}\;\mathrm{of}\;\mathrm{the}\;\mathrm{negative}\;\mathrm{control}\right)}\times 100$$

Then, 50% of the SARS-CoV-2 pseudovirus-neutralizing antibody titer (pVNT_50_) was determined by non-linear regression using GraphPad Prism 9.3.0 (GraphPad Software, Inc., San Diego, CA, USA). pVNT_50_ below the detection limit was recorded as 10 and was considered the cutoff titer.

### 2.4. Enzyme-Linked Immunospot (ELISpot) Assay

The cryopreserved PBMCs were thawed, and 2.5 × 10^5^ PBMCs/well in a 96-well plate were stimulated with 0.16 μg/mL SARS-CoV-2 M-, N-, and S-protein overlapping peptide pools (Miltenyi Biotec, San Diego, CA, USA) for 18 h at 37 °C in an atmosphere of 5% CO_2_; cells stimulated with anti-CD3 mAb (100 ng/mL; MABTECH AB, Stockholm, Sweden) were used as positive controls; cells stimulated with the medium (RPMI 1640, Lonza) alone were used as negative controls. IFN-γ spot-forming counts (SFCs) were detected using a human ELISpot^PRO^ kit (MABTECH) according to the manufacturer’s instructions. The number of spots was counted using an ImmunoSpot Counter (CTL-ImmunoSpot, Cleveland, OH, USA).

### 2.5. Statistical Analysis

Data were analyzed and plotted using GraphPad Prism 9.3.0 (GraphPad Software, Inc., San Diego, CA, USA). All datasets were tested for statistical normality, and this criterion was used to decide the appropriate (parametric or nonparametric) statistical test. The Mann–Whitney test and Wilcoxon matched-pairs signed-rank test were used to compare the differences between the experimental groups. Multiple comparisons were performed using two-way ANOVA, one-way ANOVA, and Kruskal–Wallis tests for statistical analysis (significance levels: not significant (ns): *p* > 0.05, * *p* < 0.05, ** *p* < 0.01, *** *p* < 0.001, **** *p* < 0.0001).

## 3. Results

### 3.1. Immune Responses in SARS-CoV-2-Recovered Individuals

To evaluate vaccine-induced immunity in SARS-CoV-2-recovered individuals, 35 individuals were selected for a nested case–control study fromYUMC-COVID-19-R02, a longitudinal immunogenicity follow-up study of COVID-19-recovered patients (Table 1). Eighteen individuals were vaccinated with a single dose before their fourth visit (mean = 497.9 days after ISO) (eleven with ChAdOX1, four with BNT162b2, and three with mRNA-1273). The mean number of days after vaccination at the time of blood sampling was 30.7 days (range: 9–66). Seventeen age-, sex-, and days after ISO-matched unvaccinated SARS-CoV-2-recovered individuals were selected from the YUMC-COVID-19-R02 study. The mean age of the vaccinated COVID-19-recovered individuals was 56.4 years (range: 40–71), while that of the unvaccinated individuals was 53.3 years (range: 34–70). Sixteen out of the eighteen vaccinees and fifteen out of the seventeen unvaccinated individuals were women. There were 14 and 2 cases of mild and moderate disease symptoms, respectively, and one each of asymptomatic and severe disease in vaccinated individuals based on the National Early Warning Score (NEWS). All the unvaccinated individuals had mild disease symptoms.

**Figure 1:**
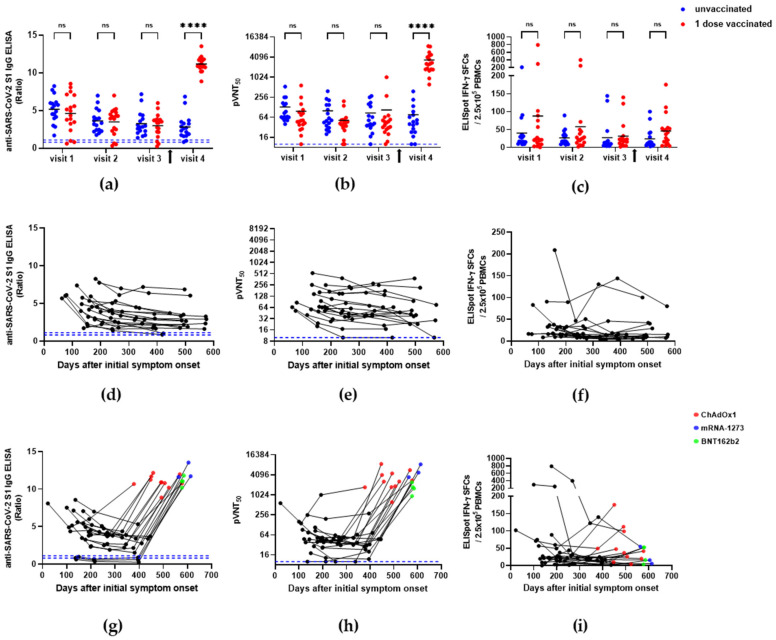
Humoral and cellular immune responses in SARS-CoV-2-recovered individuals. (**a**) SARS-CoV-2 S1 IgG ELISA ratio; (**b**) pVNT_50_ titer; (**c**) IFN-γ ELISpot counts from visit one to visit four for unvaccinated and vaccinated individuals. The arrow indicates the vaccination time in the single-dose-vaccinated group. (**d**) SARS-CoV-2 S1 IgG ELISA ratio; (**e**) pVNT_50_ titer; (**f**) IFN-γ ELISpot counts from visit one to visit four in unvaccinated individuals from the longitudinal study. (**g**) SARS-CoV-2 S1 IgG ELISA ratio; (**h**) pVNT_50_ titer; (**i**) IFN-γ ELISpot counts from visit one to visit four in single-dose-vaccinated individuals from the longitudinal study. The blue dashed line indicates the cutoff (S1-IgG = 0.8 for negative and 1.1 for positive, pVNT_50_ = 10). The significance of the differences between the unvaccinated and single-dose-vaccinated groups was determined using two-way ANOVA by Holm–Šídák’s multiple comparisons test. ns, *p* > 0.05, **** *p* < 0.0001.

The longitudinal study of the natural immunity against COVID-19 showed that anti-SARS-CoV-2 S1 binding antibody titers (S1-IgG) gradually decreased in most individuals from visit one (mean = 143.9 and 146.6 days after ISO in unvaccinated and single-dose-vaccinated groups, respectively) to visit three (mean = 331.9 and 358.7 days after ISO in unvaccinated and single-dose-vaccinated groups, respectively) (Table 1, Figure 1, left column). The mean S1-IgG was not significantly different between the unvaccinated and vaccinated groups prior to vaccination (5.2 ± 1.8 vs. 4.6 ± 2.5, 3.6 ± 1.5 vs. 3.5 ± 1.8, and 3.3 ± 1.6 vs. 3.0 ± 1.5 at visits one, two, and three, respectively; *p* > 0.05, Figure 1a, Table 2). However, the level of S1-IgG was significantly increased after a single vaccination dose (compared to the unvaccinated groups; 2.8 ± 1.6 vs. 11.2 ± 1.0; *p* < 0.0001, Figure 1a, Table 2). The mean pVNT_50_ was 131 ± 124 vs. 97 ± 133, 100 ± 98 vs. 52 ± 46, 86 ± 84 vs. 106 ± 235, and 77 ± 95 vs. 3333 ± 2322 at visits one, two, three, and four, respectively, in the unvaccinated and single-dose-vaccinated groups (Figure 1b, Table 2). The neutralizing antibody titer was also not significantly different from visits one to three between the unvaccinated and single-dose-vaccinated groups (Figure 1b); however, it was significantly increased after a single-dose vaccination (visit four) in the recovered individuals (*p* < 0.0001). The cellular immunity measured based on the levels of IFN-γ SFCs was not different between the unvaccinated and single-dose-vaccinated SARS-CoV-2-recovered individuals during all the visits (39.9 ± 49.5 vs. 87.9 ± 188.1, 26.7 ± 21.0 vs. 57.8 ± 101.0, 27.2 ± 42.6 vs. 31.6 ± 38.6, and 23.8 ± 27.6 vs. 46.0 ± 44.3 at visits one, two, three, and four, respectively; *p* > 0.05, Figure 1 right column, Table 2). Therefore, vaccination boosted humoral immunity, represented by the increase in the levels of binding (S1-IgG) and neutralizing antibodies (pVNT_50_), by 4- and 43-fold, respectively. At the same time, the counts of IFN-γ SFCs were only 1.9-fold higher in the vaccinated group than in the unvaccinated group of SARS-CoV-2-recovered individuals (Table 2).

**Figure 2:**
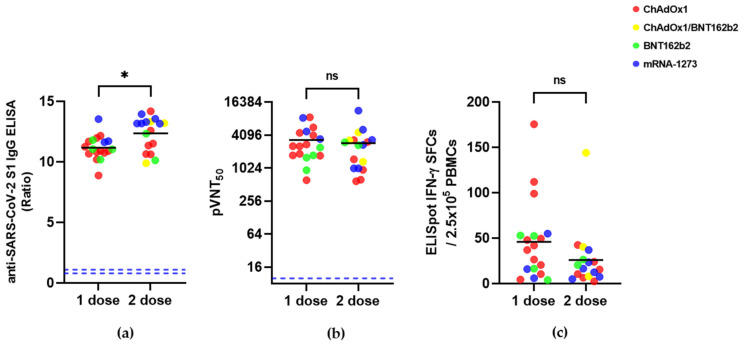
Humoral and cellular immune responses in SARS-CoV-2-recovered individuals vaccinated with a single- or double-dose COVID-19 vaccine. (**a**) SARS-CoV-2 S1 IgG ELISA ratio; (**b**) pVNT_50_ titer; (**c**) IFN-γ ELISpot counts. The blue dashed line indicates the cutoff (S1-IgG = 0.8 for negative and 1.1 for positive, pVNT_50_ = 10). *p* values were determined using the Mann–Whitney test. ns, *p* > 0.05, * *p* < 0.05.

Next, we compared the differences in humoral and cellular immunity between the single- (*n* = 18) and double-dose- (*n* = 17) vaccinated SARS-CoV-2-recovered individuals (Figure 2, Table 3). The mean age of the double-dose-vaccinated COVID-19-recovered individuals was 56.7 years (range: 23–79); among these, 12 individuals were women. Among the 17 individuals who received double-dose vaccines, 14 had mild disease, 2 were asymptomatic, and 1 had moderate disease symptoms. The mean number of days after ISO during the second dose administration was 538.3 days (range: 430–642 days). The mean number of days after the second-dose vaccination at the time of blood sampling was 72.7 days (range: 7–141). The mean S1-IgG, pVNT_50,_ and IFN-γ SFCs were 11.2 ± 1.0 vs. 12.4 ± 1.4, 3333 ± 2322 vs. 2914 ± 2591, and 46.0 ± 44.3 vs. 26.1 ± 32.8 in single- and double-dose vaccinees, respectively. Even though the binding antibody titers differed between single and double doses, the neutralizing antibody and cellular immunity were not significantly different between these regimens in SARS-CoV-2-recovered individuals (*p* > 0.05) (Figure 2, Table 3).

### 3.2. Immune Responses in SARS-CoV-2-Naïve Individuals

To evaluate vaccine-induced immunity in SARS-CoV-2-naïve individuals, 62 individuals who neither had been diagnosed with COVID-19 nor had any related symptoms were enrolled. Fifty-six individuals were vaccinated with a complete schedule of Ad26.CoV.S, ChAdOX1, BNT162b2, or mRNA-1273, and six individuals were cross-vaccinated with primary ChAdOX1 and secondary BNT162b2 doses (Table 4). According to the Korean government’s COVID-19 vaccine policy, Ad26.CoV2.S was administered to young men, and ChAdOX1 was mainly administered to individuals older than 50 years of age. Therefore, the age and sex distributions were not even in the five vaccine groups; nevertheless, the total sex ratio was similar, i.e., 30:32 in women and men (Table 4).

**Figure 3:**
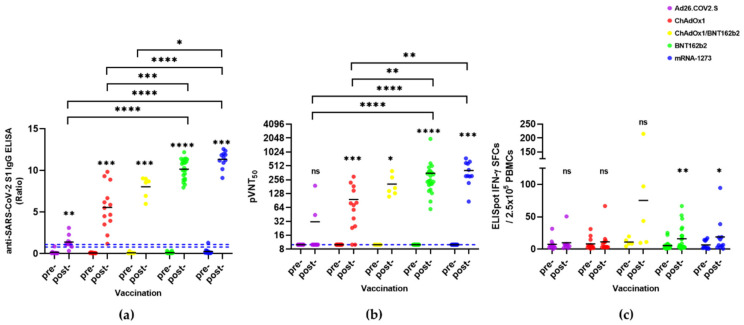
Pre- and post-vaccination humoral and cellular immunities of SARS-CoV-2-naïve participants. (**a**) SARS-CoV-2 S1 IgG ELISA; (**b**) pVNT_50_ titer; (**c**) IFN-γ ELISpot count. Wilcoxon matched-pairs signed-rank test was used to compare differences between each pre- and post-vaccination. The blue dashed line indicates the cutoff (S1-IgG = 0.8 for negative and 1.1 for positive, pVNT_50_ = 10). Kruskal–Wallis test by Dunn’s multiple comparisons test was used to compare differences between the vaccine groups. ns, *p* > 0.05, * *p* < 0.05, ** *p* < 0.01, *** *p* < 0.001, **** *p* < 0.0001.

The anti-SARS-CoV-2 nucleocapsid antibody titer evaluated in all the SARS-CoV-2-naïve individuals tested negative, suggesting that no previously infected individuals were included in the study (Supplementary Table S1). After COVID-19 vaccination, humoral immunity against SARS-CoV-2 was significantly increased in all vaccine groups except Ad26.CoV2.S (Figure 3). The S1-IgG antibody titer was the highest in mRNA-1273 (11.3 ± 1.0), followed by BNT162b2 (10.2 ± 1.2), ChAdOX1/BNT162b2 combination (8.0 ± 1.3), ChAdOX1 (5.6 ± 2.7), and Ad26.CoV2.S (1.4 ± 0.8) (Table 5). pVNT_50_ was also the highest in mRNA-1273 (406 ± 200), followed by BNT162b2 (355 ± 384), ChAdOX1/BNT162b2 combination (206 ± 111), ChAdOX1 (96 ± 90), and Ad26.CoV2.S (31 ± 56) (Table 5). Among the ChAdOX1 vaccinees, 15.4% (2/13) were negative for pVNT_50_, and most Ad26.CoV2.S vaccinees were not seroconverted in pVNT_50_ (80%, 8/10) (Figure 3a,b). Therefore, adenoviral vector vaccines induced much lower humoral immunity than mRNA vaccines (Figure 3a,b). The cellular immunity of COVID-19 vaccines Ad26.CoV2.S, ChAdOX1, and ChAdOX1/BNT162b2 in SARS-CoV-2-naïve individuals did not differ significantly between pre- and post-vaccination (*p* > 0.05). However, the IFN-γ ELISpot counts were significantly different pre- and post-vaccination (5.3 ± 6.4 vs. 15.9 ± 18.7 and 6.5 ± 6.6 vs. 18.9 ± 27.4 in BNT162b2 and mRNA-1273 vaccines, respectively; *p* < 0.05). There were no differences in the counts of IFN-γ SFCs between each group post-vaccination (*p* > 0.05).

## 4. Discussion

Here, we performed an age-, sex-, and ISO-matched nested case–control study between a single-dose-vaccinated group and an unvaccinated group of SARS-CoV-2-recovered individuals, in addition to a comparison with infection-naïve individuals. Case–control groups showed similar S1-IgG, pVNT_50_, and IFN-γ spot counts before vaccination (from visits one to three). However, the humoral immune response, especially the neutralizing antibody, was significantly increased after a single-dose vaccination compared to the unvaccinated, recovered group. Therefore, protective immunity seems to be significantly strengthened by COVID-19 vaccination in SARS-CoV-2-recovered individuals. A single dose of BNT162b2, mRNA-1273, or Ad26.COV2.S induced higher anti-S antibody titers in the recovered individuals than in the unvaccinated convalescent individuals, regardless of the vaccine type [20]. In our study comprising 18 individuals, 61% of the single-dose vaccinees were administered ChAdOX1, and the others were administered mRNA vaccines: 22% BNT162b2 and 17% mRNA-1273. Similar to the conditions observed in response to Ad26.COV2.S [20], no significant immunological differences were observed between the ChAdOX1 and mRNA vaccines, regardless of the dose, in the recovered individuals (Figure S1, Figure 2). Ebinger et al. reported that the anti-S RBD antibody titer and ACE2-binding capacity after a single dose were similar to the response seen after two doses of the BNT162b2 vaccine in previously infected healthcare workers [9]. In addition, Krammer et al. also reported no difference in anti-spike antibody response after the first and second dose of mRNA vaccine in COVID-19-recovered individuals [10]. Therefore, a single-dose regimen of either ChAdOX1 or mRNA vaccines might be suitable for these individuals.

In COVID-19-naïve individuals, the humoral immune response vastly differed according to the vaccine type. Ad26.CoV2.S induced the lowest, and mRNA-1273 induced the highest immune responses compared to the other vaccines (Figure 3). In particular, the seroconversion rate of the neutralizing antibodies was very low (20%) in the Ad26.CoV2.S vaccinees, but it was 84.6% and 100% in the ChAdOX1 vaccinees and all the other types of vaccinees, respectively (Figure 3b). These data were consistent with the previous findings that the individuals who received the complete mRNA vaccine dose were all seropositive in the SARS-CoV-2 pseudovirus neutralization test. In contrast, the seropositive rate of Ad26.CoV2.S vaccines was only 9.5% [20]. According to previous data, the seroprevalence rate of neutralizing antibodies against human adenovirus type 26 (AdHu26) showed distinct geographic distributions, such as 35.5% (407/1154) in China [21], 64.6% in South Africa, and 11.7% in the USA [22]. Unfortunately, there were no seroprevalence data for AdHu26 in Korea. We could not explain whether the preexisting AdHu26 immunity influenced the immunogenicity of Ad26.CoV2.S. Despite the low prevalence of the AdHu26 antibody, the aforementioned AdHu26 seroprevalence data did not represent seroprevalence in the entire USA; the seropositive rate of Ad26.CoV2.S in the USA was also very low. Interestingly, ChAdOX1 induced a broad range of humoral immune responses compared to either mRNA or ChAdOX1/BNT162b2 vaccines in COVID-19-naïve individuals (Figure 3a,b); a similar phenomenon was reported by Jeewandara et al. [17]. Nevertheless, ChAdOX1 induced uniform humoral immune responses in the recovered individuals, which were similar to those observed in response to mRNA-1273 or BNT162b2. Therefore, ChAdOX1 might be more effective in COVID-19-recovered individuals than in infection-naïve individuals.

We analyzed the data from pre- and post-vaccination with ChAdOX1, BNT162b2, or mRNA-1273 vaccines and visit four in the recovered individuals. The anti-S1 antibody, pVNT_50_, and IFN-γ ELISpot counts differed significantly between the unvaccinated infection-naïve group and the previously unvaccinated, infection-naïve vaccinated, and recovered vaccinated groups (Figure S2, Table S2). Therefore, the three vaccines induced significantly different immunogenicity in infection-naïve and recovered individuals. However, the small number of vaccinees is a limitation of this study.

## 5. Conclusions

A single dose of ChAdOX1, BNT162b2, or mRNA-1273 COVID-19 vaccines induced significantly high levels of anti-S1 and neutralizing antibodies in SARS-CoV-2-recovered individuals. No immunological differences were observed between single- and double-dose vaccine regimens in such cases. However, Ad26.CoV2.S and ChAdOX1 induced much lower anti-S1 and neutralizing antibody levels than BNT162b2 or mRNA-1273 vaccines in SARS-CoV-2-naïve individuals. Because ChAdOX1 has several benefits, such as low cost and stability of refrigerating temperature, it would be beneficial to administer the ChAdOX1 vaccine to the SARS-CoV-2-recovered individuals in tropical areas or low-income countries. BNT162b2 and mRNA-1273 vaccines induced significant cellular immunity in SARS-CoV-2-naïve individuals (compared to pre-vaccination), but not in the recovered individuals, even though the mean IFN-γ spot counts were increased after vaccination. These data demonstrate significant variations in the immunogenicity of the currently used COVID-19 vaccines in Korea and suggest a different vaccination strategy in infection-naïve and recovered individuals.

## Figures and Tables

**Table 1:** Characteristics of SARS-CoV-2-recovered individuals selected for the nested case–control study.

Categories	Unvaccinated Group	1-Dose-Vaccinated Group
Number	17	18
Age (range)	53.3 (34–70)	56.4 (40–71)
Sex		
Female	15	16
Male	2	2
Visit (Mean days after ISO ^1^)		
Visit 1	143.9 ± 40.8	146.6 ± 42.6
Visit 2	226.4 ± 41.2	227.6 ± 41.6
Visit 3	331.9 ± 51.3	358.7 ± 62.3
Visit 4	495.7 ± 54.8	526.7 ± 65.7
Mean days after ISO at vaccination (range)	NA ^2^	497.9 (311–594)
Mean number of days after vaccination at time of blood collection (range)	NA	30.7 (9–66)

^1^ Initial symptom onset. ^2^ Not available.

**Table 2:** Mean values of the humoral and cellular immune responses in SARS-CoV-2-recovered individuals with or without a single-dose vaccination.

Visit	SARS-CoV-2 S1 IgG ELISA (Ratio)		pVNT (pVNT_50_)		IFN-γ ELISpot (SFCs/2.5 × 10^5^ Cells)	
	Unvaccinated (*n* = 17)	Vaccinated (*n* = 18)	Unvaccinated (*n* = 17)	Vaccinated (*n* = 18)	Unvaccinated (*n* = 17)	Vaccinated (*n* = 18)
V1	5.2 ± 1.8	4.6 ± 2.5	131 ± 124	97 ± 133	39.9 ± 49.5	87.9 ± 188.1
V2	3.6 ± 1.5	3.5 ± 1.8	100 ± 98	52 ± 46	26.7 ± 21.0	57.8 ± 101.0
V3	3.3 ± 1.6	3.0 ± 1.5	86 ± 84	106 ± 235	27.2 ± 42.6	31.6 ± 38.6
V4	2.8 ± 1.6	11.2 ± 1.0	77 ± 95	3333 ± 2322	23.8 ± 27.6	46.0 ± 44.3

**Table 3:** Mean values of humoral and cellular immune responses in SARS-CoV-2-recovered individuals vaccinated with a single or double COVID-19 vaccine dose.

Measurements	1 Dose (*n* = 18)	2 Dose (*n* = 17)
SARS-CoV-2 S1 IgG ELISA (Ratio)	11.2 ± 1.0	12.4 ± 1.4
pVNT (pVNT_50_)	3333 ± 2322	2914 ± 2591
IFN-γ ELISpot (SFCs/2.5 × 10^5^ cells)	46.0 ± 44.3	26.1 ± 32.8

**Table 4:** Characteristics of SARS-CoV-2-naïve participants enrolled in the COVID-19 vaccine immunogenicity study.

Characters	Vaccine Type					
	Ad26.CoV2.S	ChAdOX1	ChAdOX1/BNT162b2	BNT162b2	mRNA-1273	Total
Number	10	13	6	22	11	62
Age	34.4 (29–39)	61.4 (58–63)	41.3 (29–48)	40.4 (25–58)	28.5 (24–57)	41.8 (24–63)
Sex						
Female	0	7	1	19	3	30
Male	10	6	5	3	8	32
Days post-vaccination at time of blood collection	33 (29–34)	33 (28–35)	31 (30–33)	32 (26–36)	30 (28–34)	32 (26–36)

**Table 5:** The pre- and post-vaccination humoral and cellular immune responses in SARS-CoV-2-naïve individuals.

Vaccines	SARS-CoV-2 S1 IgG ELISA (Ratio)		pVNT (pVNT_50_)		IFN-γ ELISpot (SFC/2.5 × 10^5^ Cells)	
	Pre	Post	Pre	Post	Pre	Post
Ad26.COV2.S	0.2 ± 0.3	1.4 ± 0.8	10	31 ± 56	7.4 ± 9.1	9.8 ± 14.5
ChAdOx1	0.1 ± 0.0	5.6 ± 2.7	10	96 ± 90	8.0 ± 8.9	11.2 ± 17.2
ChAdOx1/ BNT162b2	0.1 ± 0.1	8.0 ± 1.3	10	206 ± 111	10.9 ± 5.8	75.2 ± 85.8
BNT162b2	0.1 ± 0.1	10.2 ± 1.2	10	355 ± 384	5.3 ± 6.4	15.9 ± 18.7
mRNA-1273	0.3 ± 0.4	11.3 ± 1.0	10	406 ± 200	6.5 ± 6.6	18.9 ± 27.4

## Data Availability

The deidentified data underlying the results presented in this study may be made available upon request from the corresponding author Hosun Park, at hspark@ynu.ac.kr. The data are not publicly available in accordance with funding requirements and participant privacy.

## Supplementary Materials

The following supporting information can be downloaded at: https://www.mdpi.com/article/10.3390/vaccines10020332/s1, Figure S1: Comparison of humoral and cellular immune responses induced by three kinds of COVID-19 vaccines in SARS-CoV-2-recovered individuals vaccinated with a single dose; Figure S2: Comparison of humoral and cellular immune responses in SARS-CoV-2-naïve and -recovered infected individuals (unvaccinated and vaccinated with ChAdOX1, BNT162b2, or mRNA-1273 COVID-19 vaccines). Table S1: Anti-SARS-CoV-2 nucleocapsid antibody titers in SARS-CoV-2-naïve individuals; Table S2: Mean values of humoral and cellular immune responses of SARS-CoV-2-naïve and -recovered individuals (unvaccinated and vaccinated with ChAdOX1, BNT162b2, or mRNA-1273 COVID-19 vaccines).
