# Supplementary material for: Humoral and Cellular Responses to COVID-19 Vaccines in SARS-CoV-2 Infection-Naïve and -Recovered Korean Individuals
Source: Vaccines (Basel). 2022 Feb 18;10(2):332. doi: 10.3390/vaccines10020332 (PMC8878120; doi:10.3390/vaccines10020332)
Supplement: Supplementary file 1 [file vaccines-10-00332-s001.zip › vaccines-1579709-supplementary.pdf]

Table S1. Anti-SARS-CoV-2 nucleocapsid antibody titers in SARS-CoV-2-naïve individuals.

Vaccines	SARS-CoV-2 NCP IgG ELISA (Ratio)	
	Pre	Post
**Ad26.COV2.S**	0.07±0.02	0.08±0.04
**ChAdOx1**	0.09±0.05	0.10±0.08
**ChAdOx1/BNT162b2**	0.11±0.13	0.17±0.25
**BNT162b2**	0.10±0.07	0.12±0.07
**mRNA-1273**	0.17±0.14	0.22±0.26

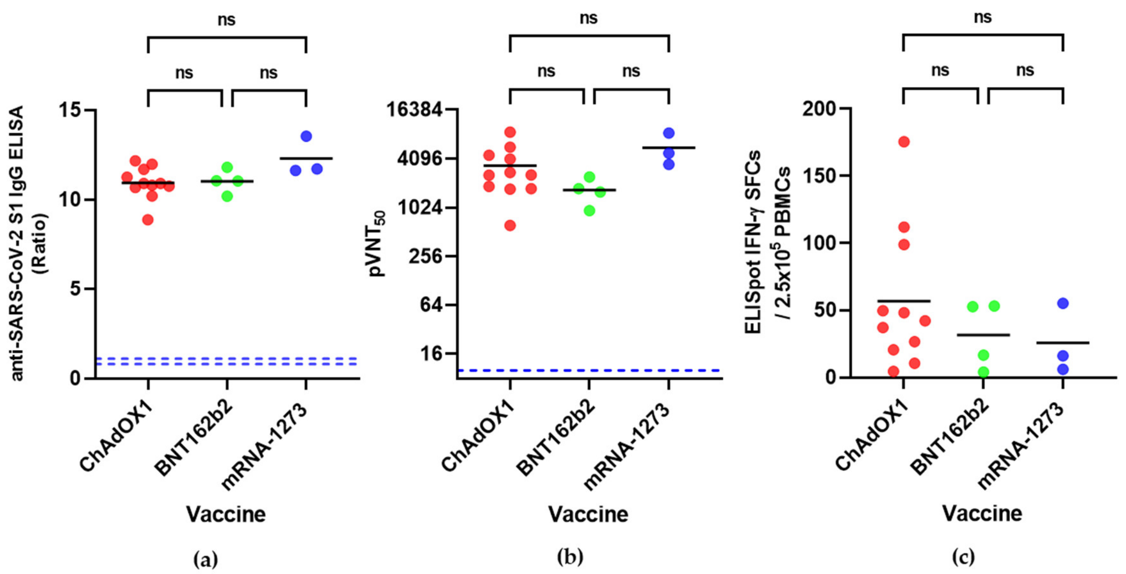

Figure S1. Comparison of humoral and cellular immune responses induced by three kinds of COVID-19 vaccines in SARS-CoV-2-recovered individuals vaccinated with a single dose. (a) SARS-CoV-2 S1 IgG ELISA ratio; (b) $pVNT_{50}$ titer; (c) IFN-γ ELISpot counts. The blue dashed line indicates the cutoff (S1-IgG=0.8 for positive and 1.1 for negative, $pVNT_{50}$ =10). Significance was tested using one-way ANOVA with Tukey's multiple comparisons or Kruskal–Wallis test by Dunn's multiple comparisons test (ns, $p > 0.05$).

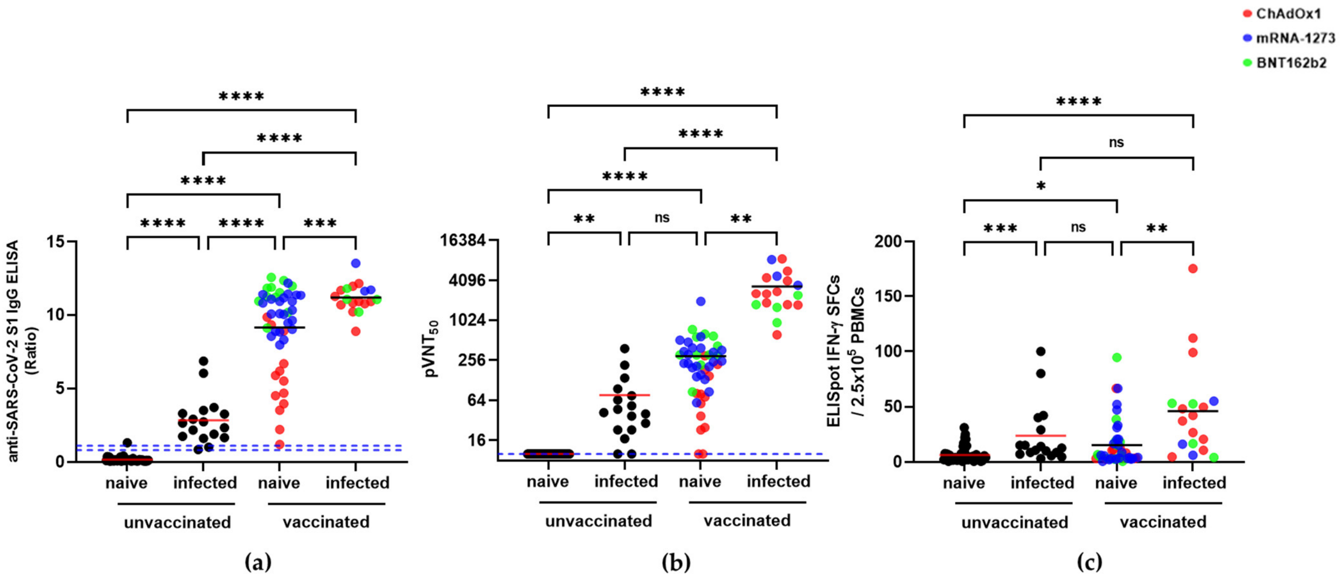

Figure S2 Comparison of humoral and cellular immune responses in SARS-CoV-2-naïve and -recovered individuals (unvaccinated and vaccinated with either ChAdOX1, BNT162b2, or mRNA-1273 COVID-19 vaccines). (a) SARS-CoV-2 S1 IgG ELISA ratio; (b) $pVNT_{50}$ titer; (c) IFN-γ ELISpot counts. The blue dashed line indicates the cutoff (S1-IgG=0.8 for negative and 1.1 for positive, $pVNT_{50}$ =10). Significance was tested using one-way ANOVA with Tukey's multiple comparisons test or Kruskal–Wallis test by Dunn's multiple comparisons test. ns, $p > 0.05$, $^{*}p < 0.05$, $^{**}p < 0.01$, $^{***}p < 0.001$, $^{****}p < 0.0001$

Table S2. Mean values of humoral and cellular immune responses of SARS-CoV-2 naïve and recovered individuals (unvaccinated and vaccinated with either ChAdOX1, BNT162b2, or mRNA-1273 COVID-19 vaccines).

SARS-CoV-2	SARS-CoV-2 IgG ELISA (Ratio)		pVNT ($pVNT_{50}$)		IFN-γ ELISpot (SFCs/2.5x10^5 cells)	
	Unvaccinated	Vaccinated	Unvaccinated	Vaccinated	Unvaccinated	Vaccinated
**Naïve**	0.2±0.2	9.2±2.9	10	294±310	6.3±7.1	15.3±20.4
**Infected**	2.8±1.6	11.2±1.0	77±95	3,333±2,322	23.8±27.6	46.0±44.3
